# Acetyl-11-keto-beta-boswellic acid ameliorates monosodium iodoacetate-induced osteoarthritis in rats: implications of HMGB1/TLR4/NF-κB and Nrf2/HO-1

**DOI:** 10.3389/fphar.2025.1694803

**Published:** 2025-12-01

**Authors:** Hagar B. Abo-Zalam, Abdallah M. Gendy, Bassam Mohamed Ali, Ghada M. Ragab, Heba Mohammed Refat M. Selim, Najat O. Hamed, Fatemah A. Alherz, Asmaa Saleh, Einas M. Yousef, Amira A. El-Gazar

**Affiliations:** 1 Department of Pharmacology and Toxicology, Faculty of Pharmacy, October 6 University, Giza, Egypt; 2 Department of Biochemistry, Faculty of Pharmacy, October 6 University, Giza, Egypt; 3 Pharmacology and Toxicology Department, Faculty of Pharmacy, Misr University for Science and Technology, Giza, Egypt; 4 Department of Pharmaceutical Sciences, College of Pharmacy, AlMaarefa University, Diriyah, Riyadh, Saudi Arabia; 5 Research Center, Deanship of Scientific Research and Post-Graduate Studies, AlMaarefa University, Diriyah, Riyadh, Saudi Arabia; 6 Department of Pharmaceutical Sciences, College of Pharmacy, Princess Nourah Bint Abdulrahman University, Riyadh, Saudi Arabia; 7 College of Medicine, Alfaisal University, Riyadh, Saudi Arabia

**Keywords:** osteoarthritis, 3-O-acetyl-11-keto-β-boswellic acid, miR-34a-5p, high-mobility group box 1, necroptosis, Nrf2

## Abstract

**Introduction:**

Osteoarthritis (OA) is a prevalent joint disorder marked by chronic inflammation and degradation of cartilage. The shielding effect of 3-O-acetyl-11-keto-β-boswellic acid (AKBA) has been confirmed in many inflammatory disorders. However, the full underlying mechanistic perspective of AKBA against OA remains unexplored. Thus, the objective of this work is to identify additional unexamined modulatory signals of AKBA against monosodium iodoacetate (MIA)-induced OA and the early prevention of irreversible cartilage damage.

**Methods:**

Male Wistar rats were allotted into three groups (n = 9): sham, MIA-OA, and MIA + AKBA250. Three mg of MIA was injected intra-articularly into the right knee joints of rats to induce OA on day 0. All treatments were given orally, daily, starting on the 3rd day after MIA injection and continuing until the 14th day of the experiment.

**Results:**

AKBA250 treatment alleviated edema completely, achieving nearly basal records of the right knee diameter. AKBA250 treatment enhanced macroscopic and microscopic findings and normalized the modified Mankin and OARSI scoring system. Moreover, AKBA restored cartilage matrix homeostasis by suppressing the catabolic enzyme matrix metalloproteinase-13 (MMP-13), upregulating tissue inhibitor of metalloproteinase-1 (TIMP-1) and the chondrogenic marker SRY-box transcription factor 9 (SOX9), and reducing the serum level of the cartilage degradation biomarker C-telopeptide of type II collagen (CTX-II). The contents of high-mobility group box 1 (HMGB1), toll-like receptor 4 (TLR4), nuclear factor-kappa B (NF-κB), and tumor necrosis factor-α (TNF-α) were substantially suppressed in the AKBA250-treated group. AKBA250 significantly boosted the nuclear factor erythroid-2-related factor 2 (Nrf2) and heme oxygenase-1 (HO-1) levels and restored the oxidant/antioxidant equilibrium disrupted by MIA. In addition, AKBA250 prominently curbed the protein expression of p-RIPK1, p-RIPK3, and p-MLKL. Furthermore, AKBA250 exhibited downregulation of miR-34a-5p and miR-146a expressions.

**Conclusion:**

Together, AKBA demonstrated a protective function in OA by inhibiting inflammatory signaling through the HMGB1/TLR4/NF-κB pathway, augmenting the cytoprotective Nrf2/HO-1 pathway, and regulating necroptosis signaling cascades.

## Graphical Abstract

**Figure:**
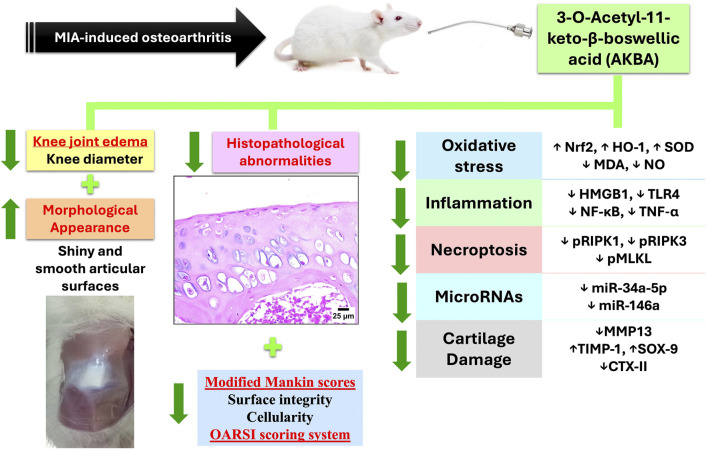

## 1 Introduction

Osteoarthritis (OA) is a chronic degenerative disease that worsens with time and age. OA affects structures throughout the joints, causing structural deterioration and functional abnormalities (Motta et al., 2023). Knee OA can be achieved by intra-articular injection of monosodium iodoacetate (MIA) (de Sousa Valente, 2019). The MIA model is extensively utilized in the field of pain research and serves as a reliable method for assessing the effectiveness of OA treatment strategies (Lampr et al., 2014). MIA is a chemical toxin that acts by suppressing the function of glyceraldehyde 3-phosphate dehydrogenase in chondrocytes, thus leading to the degradation of the glycolytic pathway (Muley et al., 2017). The primary impact of administering MIA through intra-articular injection is the induction of chondrocyte cell death, which primarily affects the joint area. This process ultimately results in cartilage degradation and concomitant alterations in the underlying subchondral bone (Bryk et al., 2021).

Necroptosis, a regulated form of inflammatory cell death (Mifflin et al., 2020), plays a pivotal role in the pathogenesis of OA and other age-related diseases (Riegger and Brenner, 2019; Khaleque et al., 2024). Unlike apoptosis or autophagy, necroptosis is characterized by cellular swelling, plasma membrane rupture, and the release of intracellular damage-associated molecular patterns (DAMPs) into the surrounding tissues, provoking an intense inflammatory response (Galluzzi et al., 2017; Saeed et al., 2019). Among these DAMPs, high-mobility group box 1 (HMGB1) acts as a potent pro-inflammatory mediator by binding to toll-like receptor 4 (TLR4) and activating the nuclear factor-kappa B (NF-κB) pathway, which drives the production of tumor necrosis factor-α (TNF-α), interleukin-1β (IL-1β), and matrix metalloproteinases (MMPs) (Galluzzi et al., 2017; Saeed et al., 2019; Jeon et al., 2020). In OA, chondrocyte necroptosis accelerates cartilage degradation and synovial inflammation through the activation of the receptor-interacting protein kinase 1 (RIPK1)/receptor-interacting protein kinase-3 (RIPK3)/mixed lineage kinase domain-like protein (MLKL) signaling cascade, which compromises membrane integrity and amplifies local inflammatory signaling (Jeon et al., 2020). This process contributes to cartilage matrix destruction, subchondral bone remodeling, and overall joint degeneration. Therefore, targeting necroptosis-related pathways represents a promising therapeutic strategy to preserve chondrocyte viability and maintain joint homeostasis (Shi et al., 2023).

Despite these insights, the currently available pharmacological interventions for OA mainly provide palliative relief and lack the ability to impede the degeneration of articular cartilage (Bruyère et al., 2019; Wang et al., 2021). This highlights the urgent need to develop pharmacological agents that not only alleviate symptoms but also halt the progression of cartilage deterioration.

The Boswellia genus comprises a group of trees renowned for their aromatic resin, which possesses numerous pharmacological properties—notably, anti-inflammatory effects (Pilkhwal and Dhaneshwar, 2019). The anti-inflammatory action of Boswellia extract is attributed to boswellic acids (BAs), recognized as the primary active constituents of frankincense that prevent necroptosis (Sharma and Jana, 2020). 3-O-Acetyl-11-keto-β-boswellic acid (AKBA) is a pentacyclic triterpenoid extracted from *Boswellia serrata (B. serrata)* (Ahmed et al., 2020). Researchers have confirmed the antioxidant and anti-inflammatory properties of AKBA in age-related and chronic illnesses such as type 2 diabetes, various malignancies, and neurodegenerative disorders (Efferth and Oesch, 2022; Wei et al., 2020; Abd El-Aal et al., 2024; El-Gazar et al., 2024; Siddiqui et al., 2021; Khan et al., 2022). Additional research suggested that AKBA may prevent cell death in a rat model of ischemia brain injury by regulating the nuclear factor erythroid-2-related factor 2 (Nrf2) and heme oxygenase-1 (HO-1) regulatory cascade and controlling BCL2-associated X apoptosis regulator and caspase-3 expressions (Ding et al., 2014).

The anti-arthritic potential of AKBA has been validated, but only in combination with bisdemethoxycurcumin as a polyherbal formulation. This formulation demonstrated restoration of knee joint structural integrity, a reduction in cartilage degradation products, and pronounced antioxidant and anti-inflammatory activities (Orhan et al., 2022).

Considering the previous study, the efficacy of AKBA alone as a single pure component against MIA-induced OA requires further elucidation. In this context, the present study focuses on key pathogenic signaling pathways implicated in OA, including the inflammatory HMGB1/TLR4/NF-κB axis; cartilage matrix regulatory markers such as matrix metalloproteinase-13 (MMP-13), tissue inhibitor of metalloproteinases-1 (TIMP-1), and SRY-box transcription factor 9 (SOX9); the antioxidant Nrf2/HO-1 pathway; and the necroptotic RIPK1/RIPK3/MLKL cascade. Moreover, post-transcriptional regulation via microRNAs (miR-34a-5p and miR-146a) was investigated to provide deeper insight into the mechanistic actions of AKBA.

## 2 Materials and methods

### 2.1 Drugs and chemicals

MIA and AKBA were obtained from Sigma-Aldrich (St Louis, MO, United States). All other chemicals used in this study were of high analytical grade.

### 2.2 Animals and their handling

Twenty-seven male Wistar rats, 8–10 weeks old and weighing between 180 and 200 g, were obtained from the Animal House Colony of the National Research Center, Giza, Egypt, and housed in the October 6 University animal house under carefully monitored conditions of 22 °C ± 2 °C and a 12-h light/dark cycle.

Ethical approval of this study was granted by the Committee of the Faculty of Pharmacy at October 6 University (Permit number: PRE-Ph-2209032). Furthermore, the study adhered to the guidelines for the management and care of laboratory animals, as published by the US National Institutes of Health in 2011—specifically, the eighth edition.

### 2.3 Experimental design and OA induction

**FIGURE 1:**
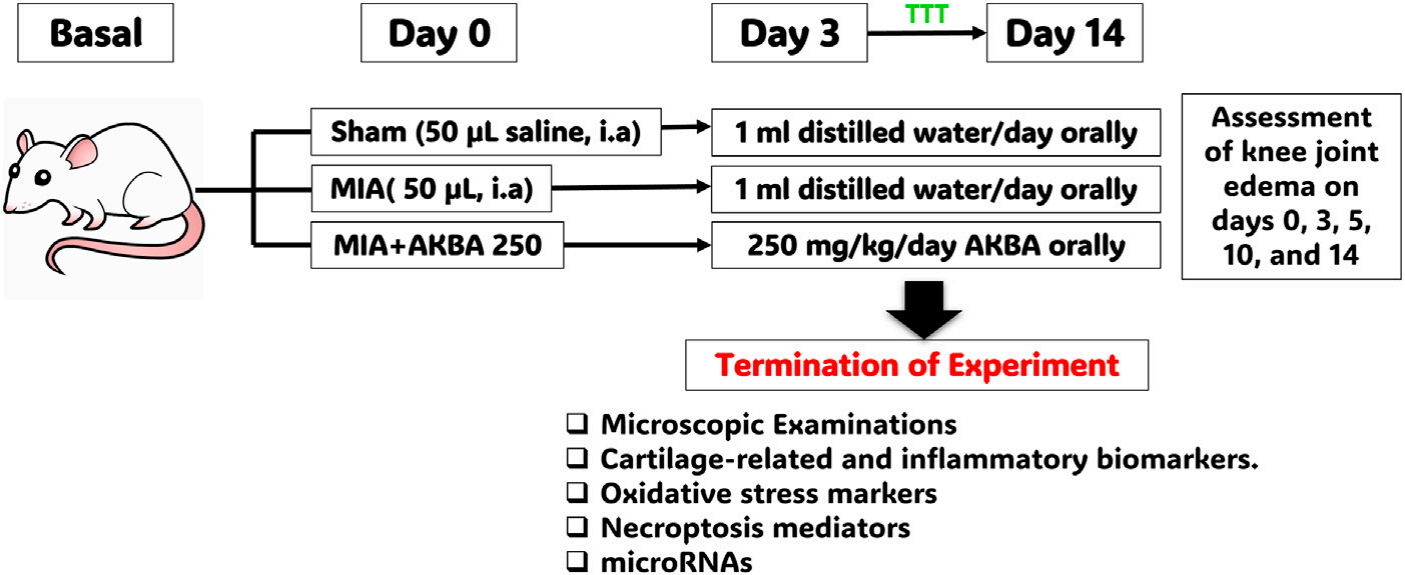
Schematic diagram of the experimental protocol. Abbreviations: AKBA, 3-O-Acetyl-11-keto-β-boswellic acid; MIA, monosodium iodoacetate.

After shaving the right knee joint under 4% isoflurane anesthesia, rats were randomly divided into three groups, with nine rats in each group, using a simple randomized method (Aldeeb et al., 2024). On day 0, the first group (sham group) was given a single intra-articular injection of 50 μL of normal saline in the right knee joint, and from the 3rd day of the experiment onward, they were administered 1 mL of distilled water orally each day until the 14th day of the experiment. The other rats were intra-articularly injected with MIA (3 mg/50 μL in normal saline) into the right knee joint on day 0 to induce knee OA (Abo-Zalam et al., 2021) and were distributed as follows: MIA group (control positive): rats were left until the 3rd day of the experiment and then were given 1 mL of distilled water orally every day until the 14th day of the experiment; MIA + AKBA250-treated group: rats were treated orally with AKBA at a dose of 250 mg/kg/day, starting from the 3rd day of MIA injection and continuing until the 14th day of the experiment. Moreover, the AKBA dose was selected based on previous studies that demonstrated the anti-inflammatory and antioxidant effects of AKBA in different experimental models (El-Gazar et al., 2024; Barakat et al., 2018; Singh et al., 2007). In addition, a preliminary dose-probing investigation comparing AKBA doses of 125 mg/kg and 250 mg/kg was performed to determine the most effective therapeutic dose; the detailed findings are provided in Supplementary Figure S1. A schematic diagram explaining the experimental protocol is presented in Figure 1.

### 2.4 Knee-joint edema evaluation

A digital caliper (model SL-1112, INSIZE Co., United States) was used to measure joint edema or swelling at different stages of OA progression. The right knee joint diameter was measured in mm on days 0, 3, 5, 10, and 14 of the experimental schedule.

### 2.5 Blood and tissue collection

On the 15th day of the study (Choi et al., 2019; Jaleel et al., 2020), anesthesia was induced in the animals via intraperitoneal injections of pentobarbital sodium (200 mg/kg) (Bao et al., 2022). Serum was obtained from femoral vein blood samples. After the euthanasia protocol, the right knee joints of three rats per group were harvested and immersed in 10% neutral-buffered formalin solution for microscopic examinations. The right knee joints of the remaining six rats per group were collected and kept at −80 °C for further biochemical and molecular analyses.

### 2.6 Enzyme-linked immunosorbent assay assessments

Complying with the manufacturer’s instructions, the corresponding enzyme-linked immunosorbent assay (ELISA) kits were utilized after homogenization of the frozen knee joint tissues in cold phosphate-buffered saline (PBS) supplemented with protease inhibitors for assessing the following parameters: HMGB1, TLR4, NF-κB, TNF-α, Nrf2, and HO-1 (MyBioSource, CA, United States, Cat. # MBS729203, MBS705488, MBS287521, MBS355371, MBS012148, and MBS014425, respectively). The caspase-3 ELISA kit was provided by ELK Biotechnology, WHU, CN (Cat. # ELK1528). The knee joint contents of superoxide dismutase (SOD; Cat. # SD 25 21), malondialdehyde (MDA; Cat. # MD 25 29), and nitric oxide (NO; Cat. # NO 25 33) were assessed using colorimetric commercial kits from Bio Diagnostics (Giza, Egypt). Furthermore, serum levels of crosslinked C-telopeptide of type II collagen (CTX-II) were assessed using a rat ELISA kit supplied by MyBioSource, CA, United States (Cat. # MBS265691).

### 2.7 Quantitative real-time polymerase chain reaction

The RNeasy Mini Kit (Cat. # 74104, QIAGEN, Valencia, CA, United States) was used to extract RNA from the right knee joint tissues. A NanoDrop^®^ (ND)-1000 Spectrophotometer (NanoDrop Technologies, Inc., Wilmington, NC, United States) was used to measure RNA samples and determine their purity. Reverse transcription was conducted using the RevertAid Reverse Transcriptase RT-PCR Kit (Cat. # EP0441, Invitrogen; Thermo Fisher Scientific, Inc., Waltham, MA, United States), according to the manufacturer’s instructions. Quantitative real-time polymerase chain reaction (qRT-PCR) was conducted using the QuantiTect SYBR Green PCR Kit (Cat. # 204141, QIAGEN, Valencia, CA, United States). The primer sequences utilized in this study were obtained from PubMed-published sequences of TIMP-1 (Rabie et al., 2023), miR-146a (El-Gazar et al., 2022), miR-34a-5p, U6 (Elbaz et al., 2024), SOX9 (Liu et al., 2013), MMP-13 (Ma et al., 2018), and β-actin (Fahim et al., 2019) genes, as listed in Table 1. The housekeeping gene (β-actin gene) was utilized as a constitutive control to aid in the normalization of TIMP-1. The levels of miR-34a-5p and miR-146a were standardized against U6, which acted as the control. As fold-change, all values were estimated with respect to β-actin or U6 using the 2^−ΔΔCT^ method (Livak and Schmittgen, 2001).

**TABLE 1:** PCR primers.

Parameter	Primer sequences (5′→ 3′)
TIMP-1	F: TCAAGGCTATGCACACTGGT R: CACTATGGTCTTTTCAATGCCTAA
miR-34a-5p	F: GCAGTGGCAGTGTCTTAG R: GGTCCAGTTTTTTTTTTTTTTTACAAC
miR-146a	F: CTGAGAACTGAATTCCA R: GAG CAG GCT GGA GAA
U6	F: GCTTCGGCAGCACATATACTAAAAT R: CGCTTCACGAATTTGCGTGTCAT
SOX-9	F: GAACGCACATCAAGACGGAG R:TCTCGTTGATTTCGCTGCTC
MMP-13	F:TCCCAGGAATTGGTGATAAAGTAGA R: CTGGCATGACGCGAACAATA
β-actin	F: CATCCTGCGTCTGGACCTGG R: TAATGTCACGCACGATTTCC

### 2.8 Western blot assessments

The frozen right knee joints were homogenized in radioimmunoprecipitation assay (RIPA) buffer for Western blot analysis to determine the protein expression of p-RIPK1, p-RIPK3, and p-MLKL. In brief, the Bicinchoninic Acid (BCA) Protein Assay Kit (Cat. # 786-570, G-Biosciences, United States) was used to measure the protein content. The protein samples were separated into equal aliquots utilizing sodium dodecyl sulfate-polyacrylamide gel electrophoresis. Then, the samples were subsequently transferred onto a nitrocellulose membrane. Thereafter, the membranes were probed with primary antibodies for an extended period of time at 4 °C that selectively recognize RIPK1 (Cat. # ab72139, Abcam, CB, United Kingdom), p-RIPK1 (phosphorylated at Ser166, Cat. # 311225, Cell Signaling Technology, MA, United States), RIPK3 (Cat. # NBP1–77299, Novus Biologicals, Littleton, CO, United States), p-RIPK3 (phosphorylated at Ser232, Cat. # ab195117, Abcam, CB, United Kingdom), MLKL (Cat. # ab194699, Abcam, CB, United Kingdom), and p-MLKL (phosphorylated at Ser358, Cat. # ab187091, Abcam, CB, United Kingdom). The secondary antibody conjugated with horseradish peroxidase (Dako, Glostrup, Denmark) was incubated overnight. Subsequently, the Western Lightning Plus ECL Chemiluminescence Reagent (PerkinElmer, MA, United States) was applied, and the band signals were recorded using a ChemiDoc Imager (Bio-Rad, CA, United States). Similarly, to assess the nuclear levels of Nrf2 (Cat. # PA5-27882, Invitrogen; Thermo Fisher Scientific, Waltham, MA, United States) and NF-κB (Cat. # MA5-15160, Invitrogen; Thermo Fisher Scientific, Waltham, MA, United States), the nuclear protein extracts were prepared using the Nuclear Extraction Kit (Cat. # NBP2-29447, Novus Biologicals, Littleton, CO, United States), according to the manufacturer’s instructions, and the resulting fractions were subjected to Western blot analysis under the same conditions. For nuclear proteins, band intensities were normalized to histone H3 (Cata # PA5-16183, Invitrogen; Thermo Fisher Scientific, Waltham, MA, United States) to ensure equal nuclear loading. The p-NF-κB was normalized to its corresponding total NF-κB protein (Cat. # 51-0,500,; Invitrogen; Thermo Fisher Scientific, Waltham, MA, United States) to accurately assess the relative activation levels.

### 2.9 Histopathological assessment

The right knee joints of three rats from each group were submerged in a 10% neutral buffered formalin solution for 48 h. The decalcification process was carried out using Cal-Ex^TM^ II Fixative/Decalcifier for 20 days (Fisher Chemical^TM^ Scientific, Waltham, MA, United States). The joints were dissected and assessed at various levels with serial step sections at the mid-sagittal location. The specimens were subsequently subjected to ethanol serial dilutions, followed by xylene cleaning, infiltration with an embedding medium specifically designed for paraplast tissue, and, ultimately, embedding. Fibrous connective tissue staining was performed on thin slices (4 μm–6 μm) using hematoxylin and eosin (H&E) stain. The captured H&E photomicrographs were further subjected to the modified Mankin scoring system (Moody et al., 2012), which is a histological assessment of cartilage degeneration using scores for surface integrity (0–10) and cellularity (0–4). Furthermore, safranin O stain was used to determine cartilage matrix repair and graded using the modified Mankin scoring system for matrix staining (0–5) and the Osteoarthritis Research Society International (OARSI) scoring system, as previously fully described (Pritzker et al., 2006; Gerwin et al., 2010). The histopathological scoring (modified Mankin and OARSI) was performed independently by a pathologist blinded to the experimental groups (Aldeeb et al., 2024).

### 2.10 Statistical analysis of data

The statistical analysis was conducted using GraphPad Prism software, version 9 (GraphPad Software, Inc., San Diego, CA, United States). The Shapiro–Wilk test was used to evaluate the normality of the data (Marusteri and Bacarea, 2010). Data that passed the normality test were further analyzed. Differences in knee joint diameter were evaluated using two-way repeated-measures analysis of variance (ANOVA) with one factor repetition (time: day; n = 9), while one-way ANOVA followed by Tukey’s *post hoc* test was applied to ELISA, PCR, and Western blot data (Thomas and Zumbo, 2012). The Kruskal–Wallis test was used to analyze the non-parametric data (Rizk, 2023), including the surface integrity score and cellularity score, followed by Dunn’s *post hoc* test. Data are presented as the mean ± standard deviation (SD), and a *p*-value <0.05 was considered statistically significant.

## 3 Results

### 3.1 Effect of AKBA on MIA-induced knee joint edema

**FIGURE 2:**
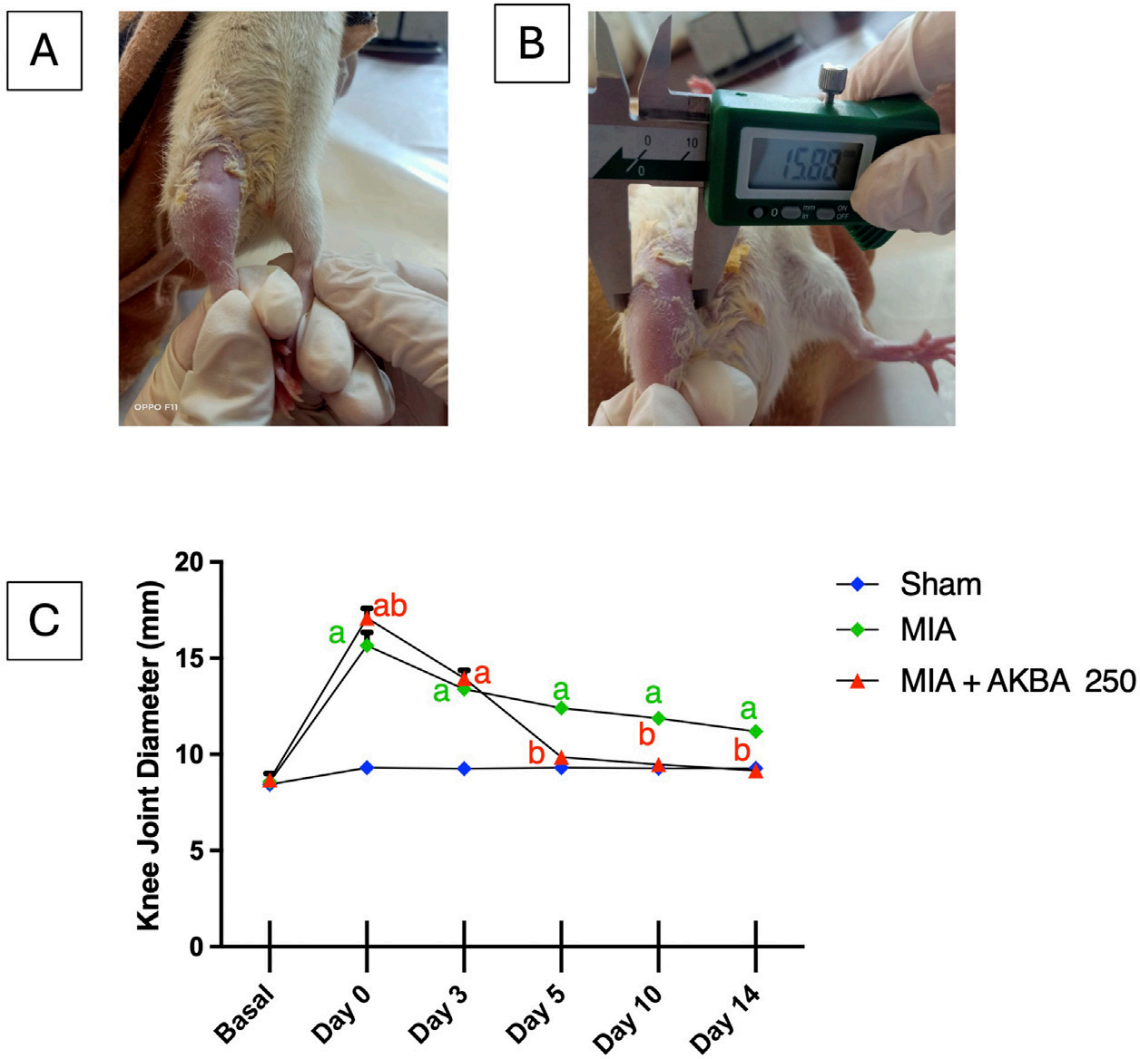
Effect of AKBA on MIA-induced knee joint edema. **(A)** Knee joint after MIA injection, **(B)** digital caliper assessment of knee joint, and **(C)** knee joint diameter records in mm. The symbol (a) reflects a significant difference from the sham group, while (b) reflects a significant difference from MIA-only treated group. Data are reported as the mean ± SD, with statistical analysis conducted using two-way repeated measures ANOVA with one factor repetition (time: day; n = 9); *p* < 0.05 was deemed significant. Abbreviations: AKBA, 3-O-Acetyl-11-keto-β-boswellic acid; MIA, monosodium iodoacetate; SD, standard deviation.

As depicted in Figure 2, all rats injected with MIA displayed a substantial increase in knee diameter on day 0 compared to their baseline values and the values of the sham group. Additionally, the MIA-exposed rats experienced enduring edema on days 3, 5, 10, and 14 of the experiment compared to the sham group. Furthermore, the rats treated with MIA + AKBA250 exhibited complete recovery from edema, attaining nearly basal values of right knee diameter on day 5 of the experimental period.

### 3.2 Impact of AKBA treatment on MIA-provoked morphological alterations of extra-articular tissues

**FIGURE 3:**
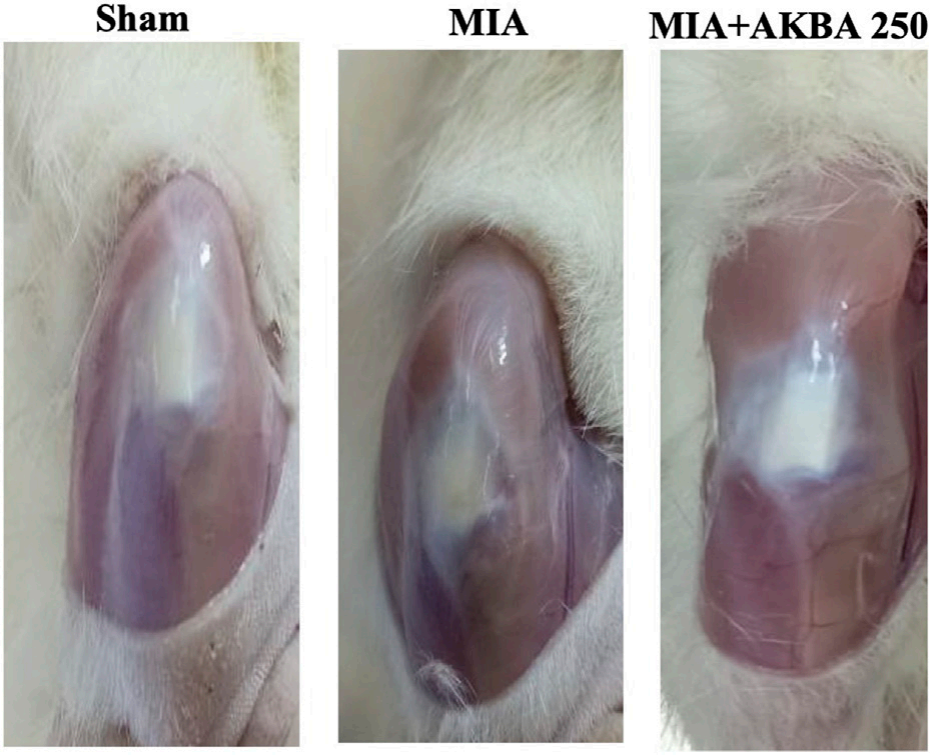
Impact of AKBA on MIA-provoked morphological alterations of extra-articular surface. The sham group’s extra-articular surface looked normal; they had shiny, smooth articular surfaces. In contrast, rats with MIA-induced OA showed a dull yellowish discoloration in their articular surfaces. The articular surfaces of the rats treated with AKBA250 were smooth and shiny. Abbreviations: AKBA, 3-O-Acetyl-11-keto-β-boswellic acid; MIA, monosodium iodoacetate.

The findings regarding the joint’s edema were further confirmed by morphological examination of the extra-articular surface on the 15th day of the query. The extra-articular surface of the sham group appeared normal, shiny, and smooth. On the contrary, the extra-articular surface of MIA-induced OA rats exhibited dull yellowish discoloration. The results also revealed the ability of AKBA250 treatment to alleviate the damaging effect of MIA, displaying a smooth and shiny articular surface that is, to some degree, similar to that of the sham rats (Figure 3).

### 3.3 Histopathological examination, modified Mankin, and OARSI scoring

The sham group showed (A) normal histological structure of the articular surface. In comparison, the MIA-related photomicrographs revealed (B) severe destruction and roughness of the articular surface (black arrow). Conversely, treatment with (C) AKBA250 mg/kg prevented the destructive effects of MIA, saved the knee joint structure, and displayed normal histological structural preference of the articular surface (H&E stain).

**FIGURE 4:**
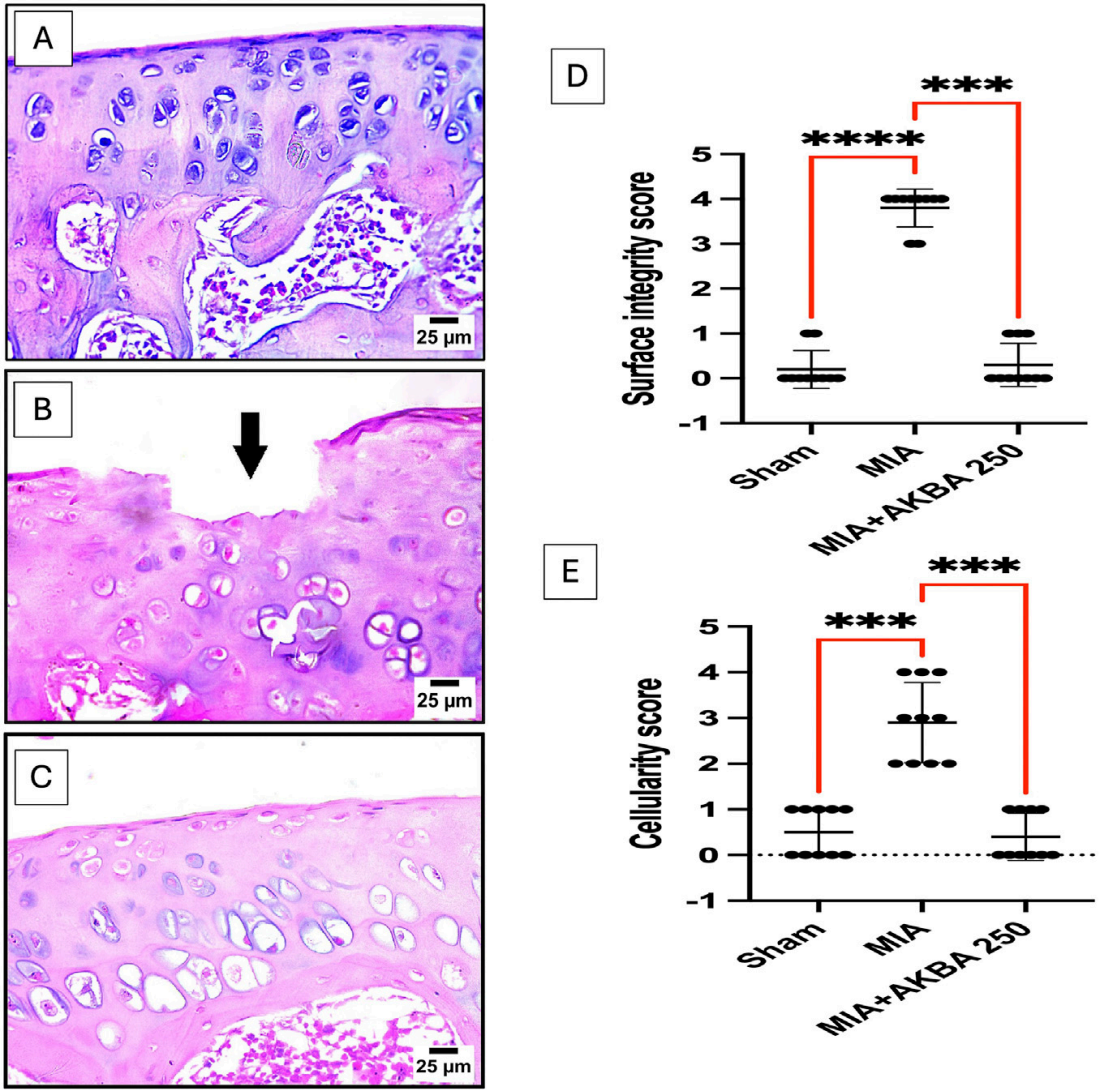
Photomicrographs of knee-joint tissue sections of the **(A)** sham group, **(B)** MIA group, and **(C)** MIA + AKBA250 group, stained with H&E and changes in **(D)** the surface integrity score and **(E)** the cellularity score caused by MIA, as measured by the modified Mankin scoring. Data are reported as the mean of 10 microscopic fields ±SD, with statistical analysis conducted using the Kruskal–Wallis test, followed by Dunn’s *post hoc* test. The number of asterisks “*” indicates the strength of significance as follows: *p < 0.05, **p < 0.01, ***p < 0.001, and ****p < 0.0001. Abbreviations: AKBA, 3-O-Acetyl-11-keto-β-boswellic acid; H&E, hematoxylin and eosin; MIA, monosodium iodoacetate.

Regarding the modified Mankin grading system, normal modified Mankin scores were recorded in the sham-operated group. On the other hand, the modified Mankin grading scoring system recorded degenerative/destructive values, which were represented by the highest values of (D) surface integrity and (E) cellularity scores. Fortunately, treatment with AKBA250 showed surface integrity and cellularity scores comparable to those of the sham-operated group. All the histopathological findings and modified Mankin scoring records are shown in Figure 4.

**FIGURE 5:**
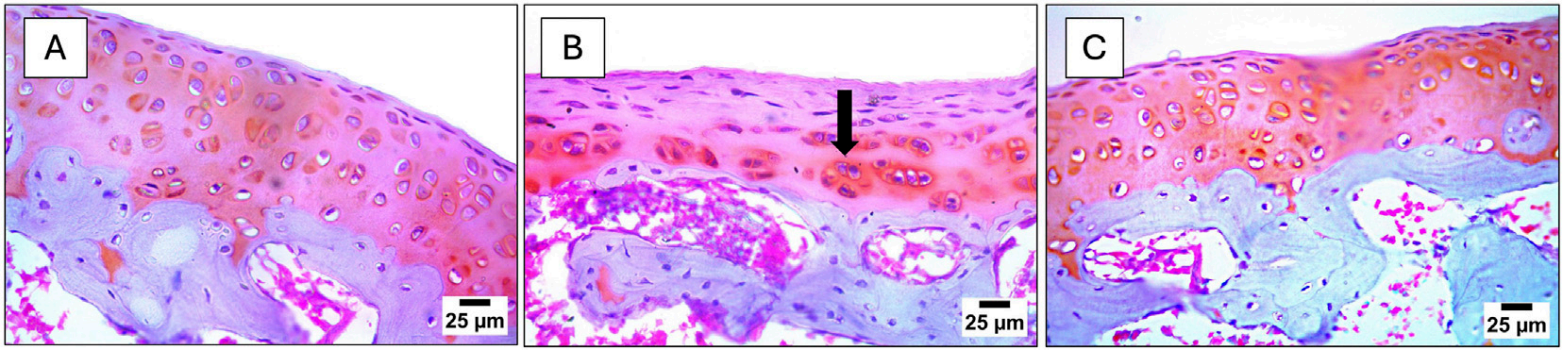
Photomicrographs of knee joint tissue sections stained with safranin O stain. **(A)** Sham group, **(B)** MIA group, and **(C)** MIA + AKBA250 group. Abbreviations: AKBA, 3-O-Acetyl-11-keto-β-boswellic acid; MIA, monosodium iodoacetate.

Moreover, safranin O stain was used to determine the effect of AKBA on proteoglycan loss in the articular surface after OA induction (Figure 5). The photomicrograph of the (B) MIA-untreated group presented a moderate loss of proteoglycan in the articular surface (black arrow), and the matrix staining score was 2, reflecting a moderate reduction compared to the normal histological structure of articular surface and the normal matrix staining score of the (A) sham group. AKBA250 showed an apparently normal histological structure of the articular surface and prevented proteoglycan loss, with a normal matrix staining score compared with that of the MIA-only injected group. Using the OARSI scoring system, the MIA group showed cartilage degradation (grade 2), relevant to the sham group, which presented a normal matrix architecture (grade 0). On the other hand, AKBA250 treatment decreased cartilage loss (grade 0) compared to the cartilage preference of the OA group.

### 3.4 Effect of AKBA on MIA-induced changes in knee joint MMP-13, TIMP-1, and SOX9 expression and serum CTX-II levels

**FIGURE 6:**
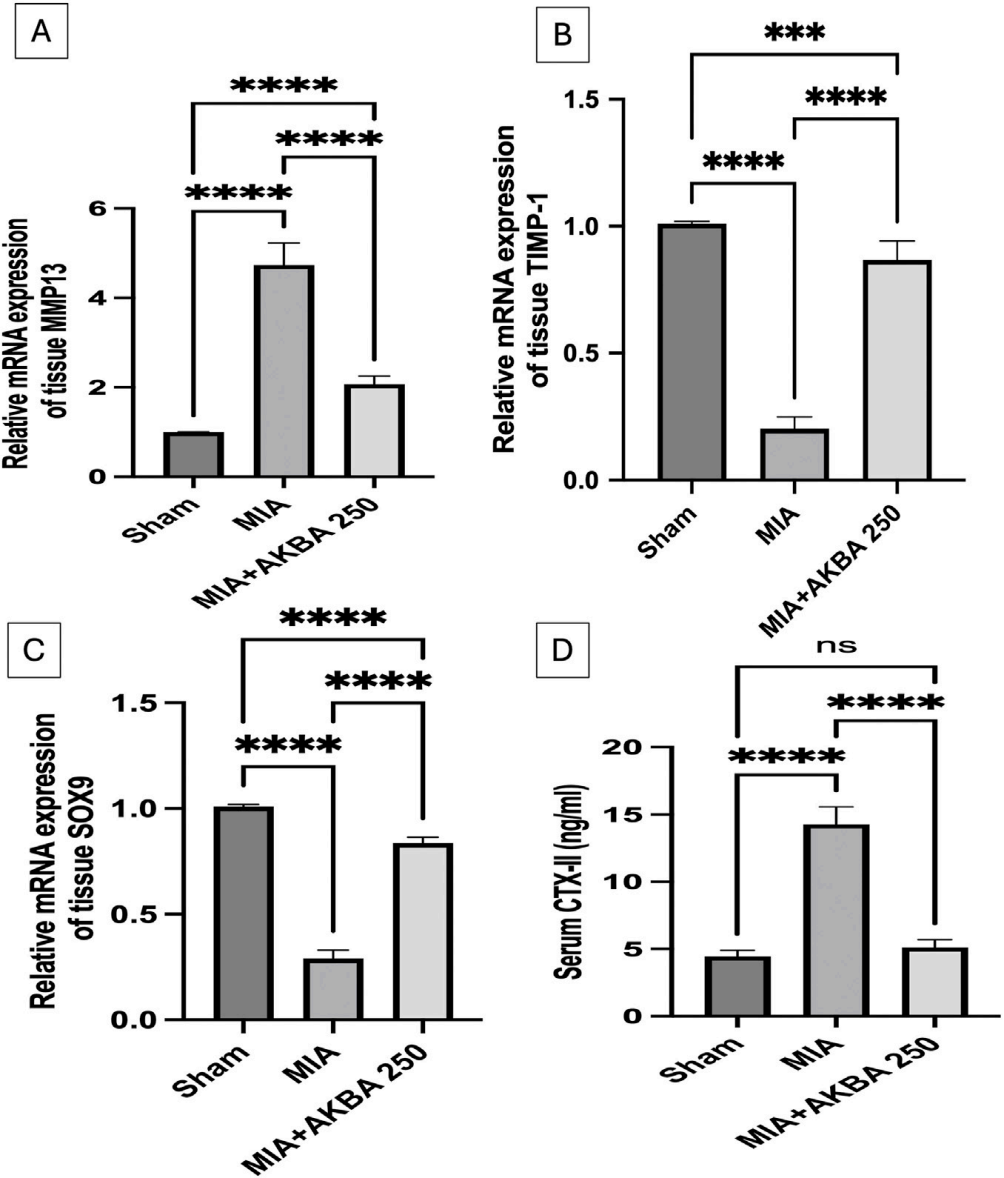
Effect of AKBA on MIA-induced changes and knee joint **(A)** MMP-13, **(B)**TIMP-1, **(C)** SOX9 expression, and serum **(D)** CTX-II levels. Data are reported as the mean (n = 6) ± SD, with statistical analysis conducted using one-way ANOVA followed by Tukey’s *post hoc* test. The number of asterisks “*” above the columns indicates the strength of significance as follows: *p < 0.05, **p < 0.01, ***p < 0.001, and ****p < 0.0001. Abbreviations: AKBA, 3-O-Acetyl-11-keto-β-boswellic acid; CTX-II, crosslinked C-telopeptide of type II collagen; MIA, monosodium iodoacetate; MMP-13, matrix metalloproteinase-13; SD, standard deviation; SOX9, SRY-box transcription factor 9; TIMP-1, tissue inhibitor of metalloproteinase-1.

MIA administration markedly disturbed cartilage matrix homeostasis by upregulating (A) MMP-13 expression, a major collagen-degrading enzyme, by 4.7 fold [F (2, 15) = 6.11, *p* < 0.0001] and reducing (B) TIMP-1 expression by 79.8% [F (2, 15) = 287.6, *p* < 0.0001] and (C) SOX9 expression by 71% [F (2, 15) = 2.313, *p* < 0.0001]. Consequently, the serum levels of (D) CTX-II, a biochemical marker of type II collagen degradation, were markedly increased by 3.2-fold [F (2, 15) = 196.3, *p* < 0.0001] relative to the sham group. Interestingly, treatment with AKBA250 reversed these detrimental alterations by suppressing MMP-13 expression, significantly elevating TIMP-1 and SOX9 levels, and reducing CTX-II serum levels (Figure 6).

### 3.5 Effect of AKBA against MIA-induced inflammatory perturbations

**FIGURE 7:**
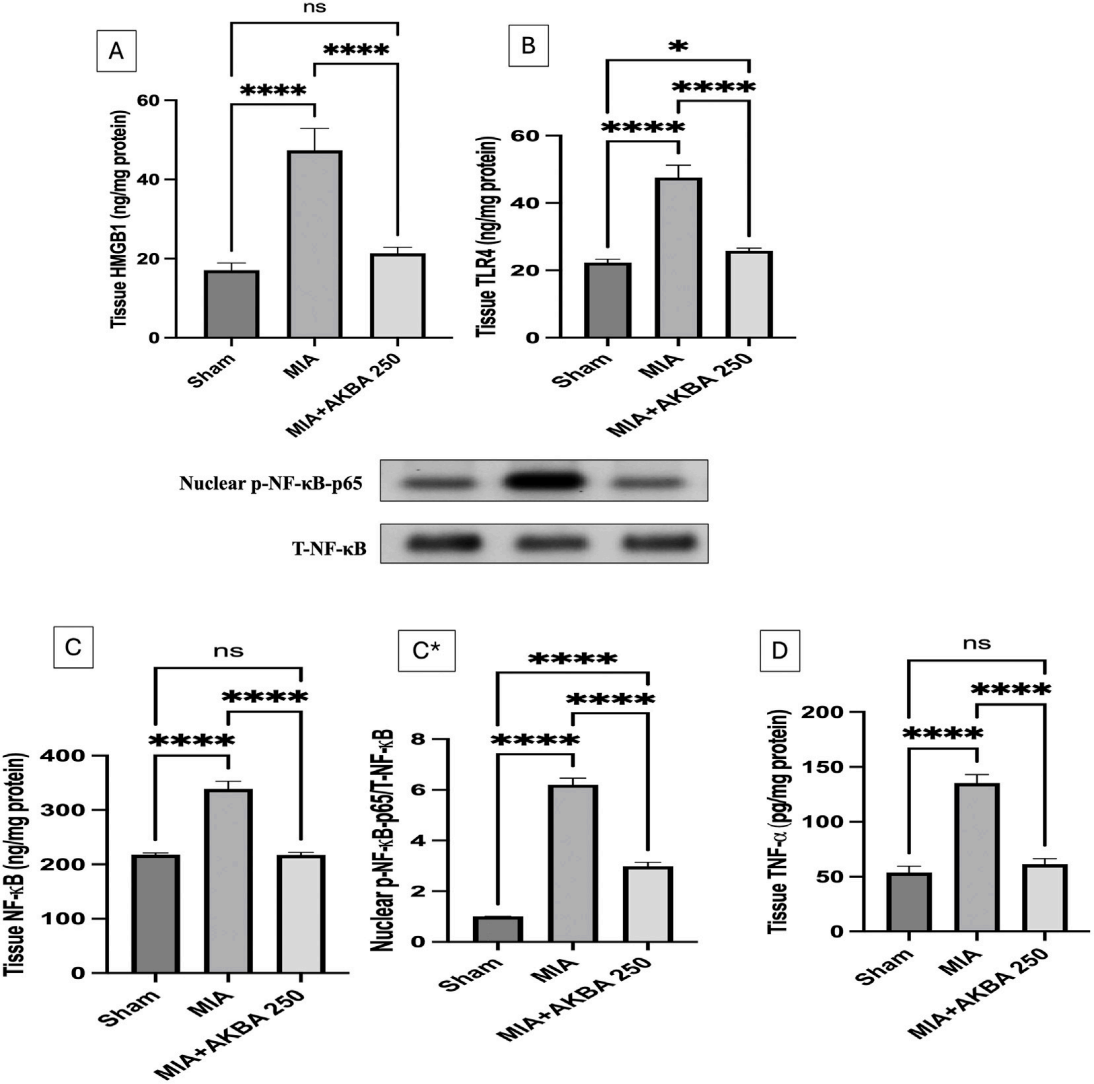
Effect of AKBA on MIA-induced changes in knee joint contents and/or expression levels of **(A)** HMGB1, **(B)** TLR4, **(C)** NF-κB, (C*) nuclear p-NF-κB–p65, and **(D)** TNF‐α. Data are reported as the mean (n = 6) ± SD, with statistical analysis conducted using one-way ANOVA followed by Tukey’s *post hoc* test. The number of asterisks “*” above the columns indicates the strength of significance as follows: *p < 0.05, **p < 0.01, ***p < 0.001, and ****p < 0.0001. Abbreviations: AKBA, 3-O-Acetyl-11-keto-β-boswellic acid; HMGB1, high mobility group box 1; MIA, monosodium iodoacetate; NF-κB, nuclear factor kappa B; nuclear p-NF-κB-p65, phosphorylated nuclear factor kappa Bp65 at serine 536; SD, standard deviation; TLR4, toll-like receptor 4; TNF-α, tumor necrosis factor-α.

The MIA-exposed rats had significantly higher (A) HMGB1 (2.7-fold) [F 2, 15) = 102.6, *p* < 0.0001], (B) TLR4 (2.1-fold) [F (2, 15) = 77.82, *p* < 0.0001], (C) NF-κB (1.5-fold) [F (2, 15) = 139.0, (*p* < 0.0001)], (C*) nuclear p-NF-κB-p65 (6.5-fold) [F (2, 6) = 0.9973, *p* < 0.0001], and (D)TNF‐α (2.5-fold) [F (2, 15) = 201.2, *p* < 0.0001] than the sham rats. Treatment with AKBA250 significantly attenuated the previous alterations, restoring the HMGB1, TLR4, NF-κB, and TNF‐α levels toward normal values and reducing NF-κB p65 nuclear translocation (Figure 7).

### 3.6 Effect of AKBA on the oxidative stress status and the apoptotic marker caspase-3 in MIA-induced OA in rats

**FIGURE 8:**
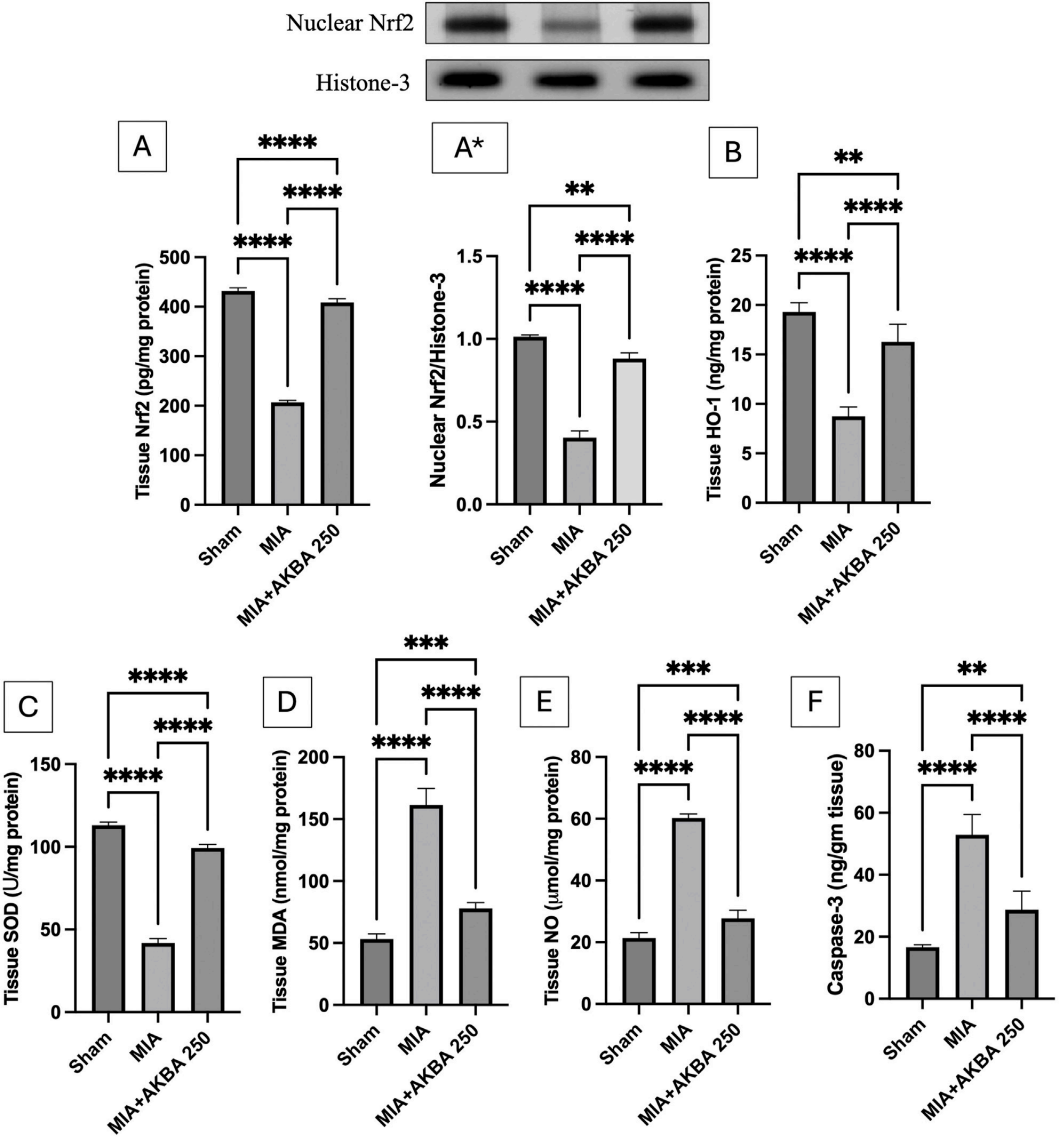
Effect of AKBA on MIA-induced changes in knee joint contents and/or expression levels of **(A)** Nrf2, (A*)nuclear Nrf2, **(B)** HO-1, **(C)** SOD activity, **(D)** MDA, **(E)** NO, and **(F)** caspase-3 Data are reported as the mean (n = 6) ± SD, with statistical analysis conducted using one-way ANOVA followed by Tukey’s *post hoc* test. The number of asterisks “*” above the columns indicates the strength of significance as follows: *p < 0.05, **p < 0.01, ***p < 0.001, and ****p < 0.0001. Abbreviations: AKBA, 3-O-Acetyl-11-keto-β-boswellic acid; HO-1, heme oxygenase-1; MDA, malondialdehyde; MIA, monosodium iodoacetate; NO, nitric oxide; Nrf2, nuclear factor erythroid 2-related factor-2; SD, standard deviation; SOD, superoxide dismutase.

As shown in Figure 8, MIA injection significantly reduced the knee joint (A) Nrf2 contents, (A*) nuclear Nrf2 expression, and (B) HO-1 contents by 52.2% [F (2, 15) = 2,268, *p* < 0.0001], 40% [F (2, 6) = 0.6078, *p* < 0.0001], and 54.7% [F (2, 15) = 108.7, *p* < 0.0001], respectively, along with the (C) SOD activity by 63% [F (2, 15) = 1,575, *p* < 0.0001] compared to that in the sham group. Oral administration of AKBA250 resulted in a significant upregulation of Nrf2 content and nuclear expression (by approximately 2-fold) and HO-1 expression (by 1.9-fold) compared with that in the MIA group. In addition, AKBA250 treatment led to a marked increase in SOD activity by 2.4‐fold compared to that in the MIA rats. Furthermore, the MIA group showed noticeably higher levels of the knee joint tissues of (D) MDA (3-fold) [F (2, 15) = 265.1, (*p* < 0.0001)], (E) NO (2.8-fold) [F (2, 15) = 672.0, (*p* < 0.0001)], and (F) caspase-3 (3.2-fold) [F (2, 15) = 77.07, (*p* < 0.0001)] than the sham group. AKBA250 treatment efficiently counteracted these effects, as manifested by reductions in MDA, NO, and caspase-3 by 51.7%, 53.9%, and 45.7%, respectively.

### 3.7 Effect of AKBA on the knee joint tissues miR-34a-5p and miR-146a gene expression in MIA-induced OA in rats

**FIGURE 9:**
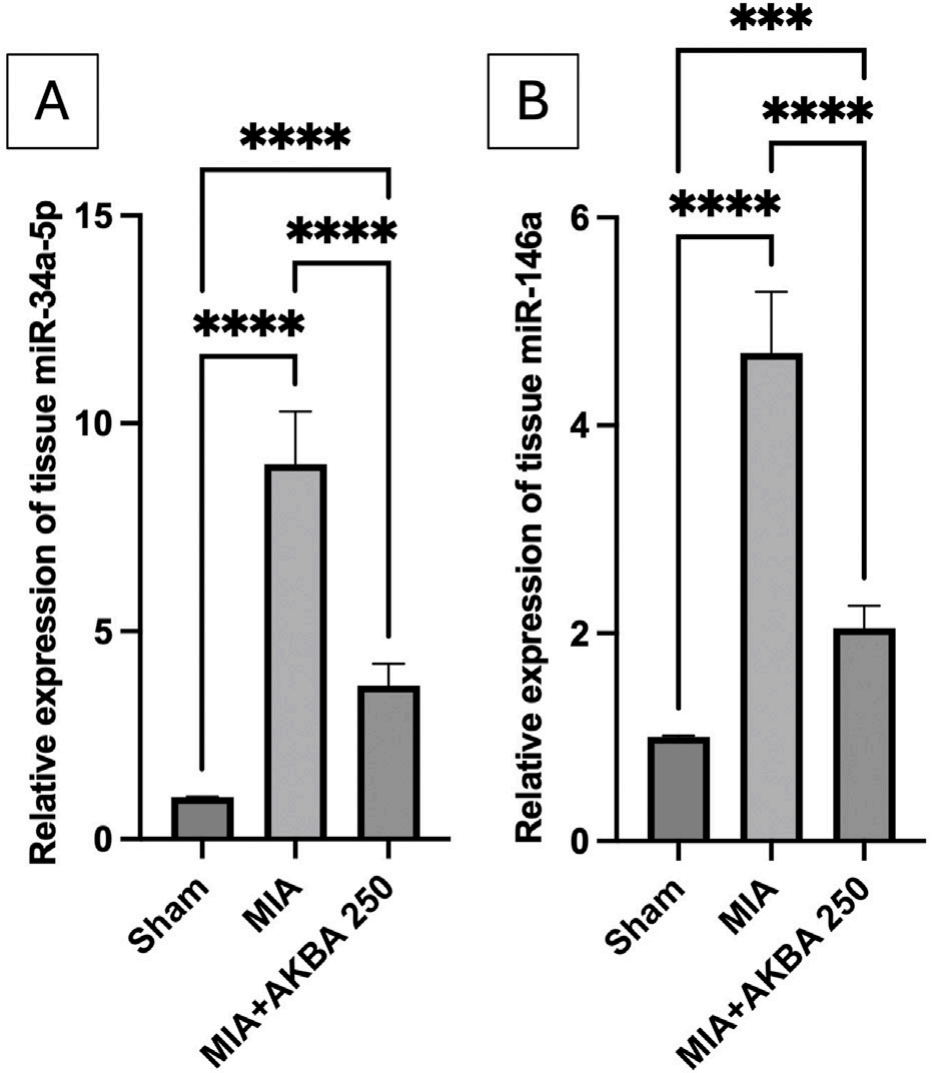
Effect of AKBA on knee joint gene expression of **(A)** miR-34a-5p and **(B)** miR-146a in MIA-induced OA in rats. Data are reported as the mean (n = 6) ± SD, with statistical analysis conducted using one-way ANOVA followed by Tukey’s *post hoc* test. The number of asterisks “*” above the columns indicates the strength of significance as follows: *p < 0.05, **p < 0.01, ***p < 0.001, and ****p < 0.0001. Abbreviations: AKBA, 3-O-Acetyl-11-keto-β-boswellic acid; MIA, monosodium iodoacetate; miR, microRNA; OA, osteoarthritis; SD, standard deviation.

Compared to the sham rats, the MIA-exposed rats experienced a prominent upregulation of the gene expression of (A) miR-34a-5p [F (2, 15) = 158.2, (*p* < 0.0001)] and (B) miR-146a [F (2, 15) = 167.0, *p* < 0.0001] by 8.9- and 4.7-fold, respectively. On the other hand, AKBA250 treatment downregulated the gene expression of miR-34a-5p and miR-146a by 59% and 56.4%, respectively, compared to that in the MIA group (Figure 9).

### 3.8 Effect of AKBA on necroptosis-related biomarkers in MIA rats

**FIGURE 10:**
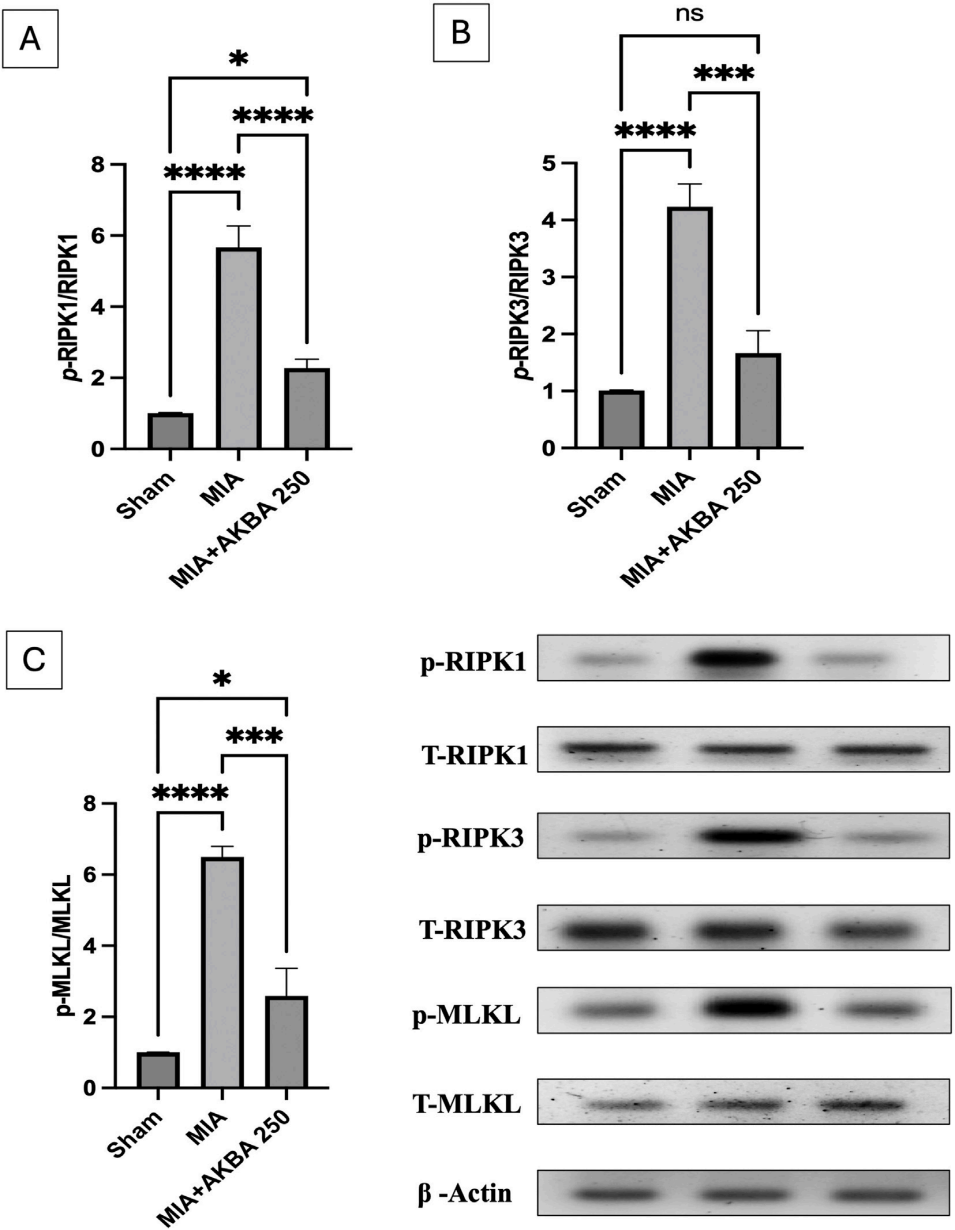
Effect of AKBA on MIA-induced changes in the knee joint protein expression of **(A)** p-RIPK1, **(B)** p-RIPK3, and **(C)** p-MLKL. Data are reported as the mean (n = 3) ± SD, with statistical analysis conducted using one-way ANOVA followed by Tukey’s *post hoc* test. The number of asterisks “*” above the columns indicates the strength of significance as follows: *p < 0.05, **p < 0.01, ***p < 0.001, and ****p < 0.0001. Abbreviations: AKBA, 3-O-Acetyl-11-keto-β-boswellic acid; MIA, monosodium iodoacetate; MLKL, mixed lineage kinase domain-like protein; RIPK1, receptor-interacting protein kinase 1; RIPK3, receptor-interacting protein kinase-3; SD, standard deviation.

The present study mainly emphasized RIPK1/RIPK3/MLKL signaling to explore the possible mechanisms by which AKBA250 alleviates knee joint tissue necroptosis. Significant increases in the protein expression of (A) p-RIPK1 [F (2, 6) = 123.1, (*p* < 0.0001)], (B) *p*-RIPK3 [F (2, 6) = 81.04, < 0.0001], and (C) p-MLKL [F (2, 6) = 105.3, *p* < 0.0001] were observed in the knee joint tissues of MIA-exposed rats to reach approximately 5.6-, 4.2-, and 6.5‐fold of the sham levels, respectively. Conversely, AKBA250 modulated the necroptosis status by suppressing p-RIPK1, p-RIPK3, and p-MLKL protein expression by 60%, 60.7%, and 60%, respectively (Figure 10).

## 4 Discussion

**FIGURE 11:**
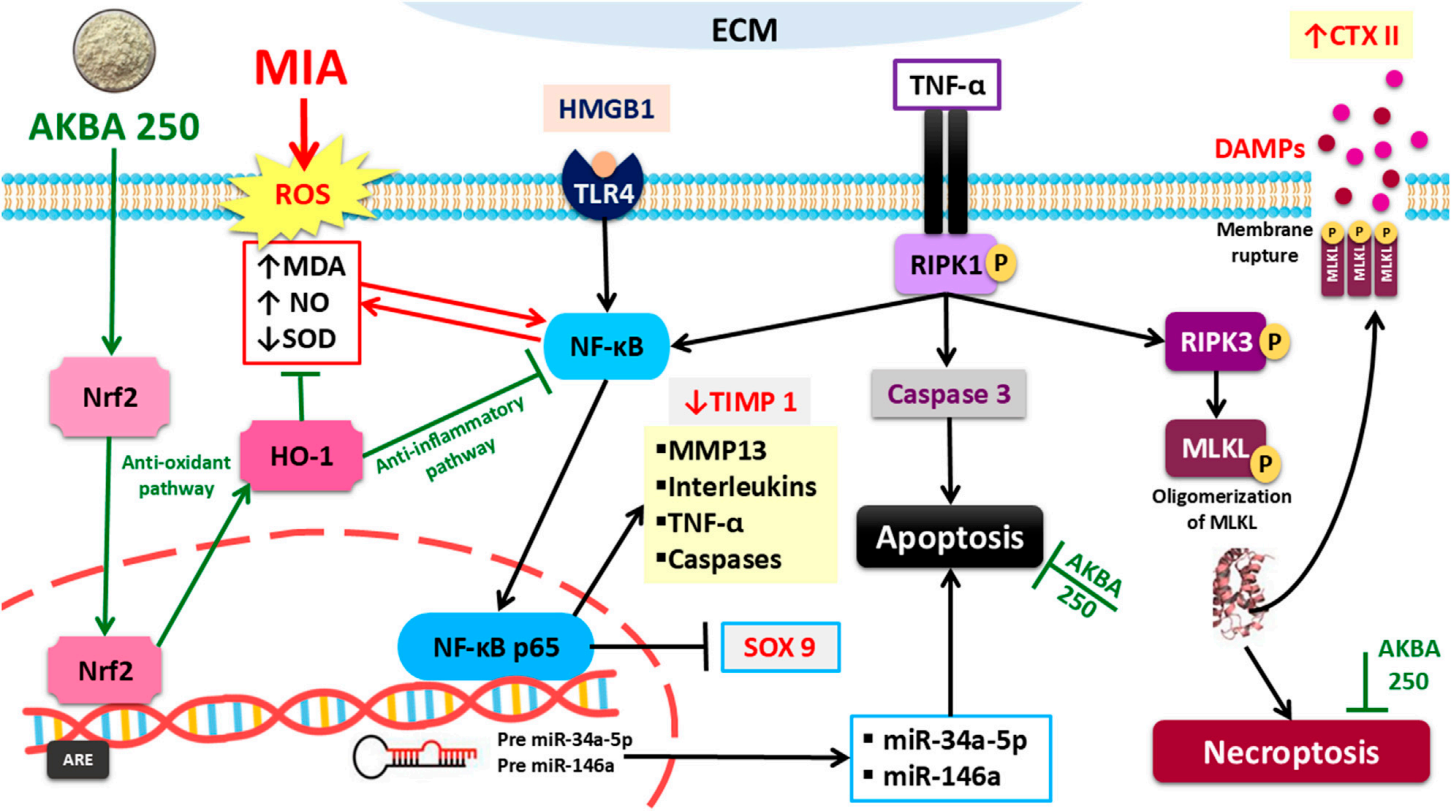
Proposed mechanistic illustration of chondroprotective effects of AKBA against MIA-induced osteoarthritis. AKBA activates the Nrf2/HO-1 antioxidant pathway, leading to enhanced cellular defense and redox balance, while concurrently inhibiting the HMGB1/TLR4/NF-κB inflammatory cascade. These effects collectively suppress necroptosis-related signaling (p-RIPK1, p-RIPK3, and p-MLKL) and downregulate microRNAs (miR-34a-5p and miR-146a). Together, these interactions contribute to restoring cartilage matrix homeostasis and mitigating osteoarthritic progression. AKBA, 3-O-Acetyl-11-keto-β-boswellic acid; CTX-II, crosslinked C-telopeptide of type II collagen; DAMPs, damage-associated molecular patterns; HMGB1, high mobility group box 1; HO-1, heme oxygenase-1; MDA, malondialdehyde; MIA, Monosodium iodoacetate; miRs, microRNAs; MLKL, mixed lineage kinase domain-like protein; NF-κB, nuclear factor kappa B; Nrf2, nuclear factor erythroid 2-related factor-2; p-RIPK1, phosphorylated receptor-interacting protein kinase 1; RIPK3, r phosphorylated-receptor-interacting protein kinase-3; SOD, superoxide dismutase; MMP-13, matrix metalloproteinase-13; SOX9, SRY-box transcription factor 9; TIMP-1, tissue inhibitor of metalloproteinase; TLR4, toll-like receptor 4; TNF-α, tumor necrosis factor-α.

This research clarifies the protective role of AKBA in modifying OA progression utilizing the MIA model after verifying various mechanistic aspects. The effectiveness of the BA derivative depends on its ability to activate the antioxidant transcription factor Nrf2, thereby reducing cellular stress and inflammatory signaling. Furthermore, a critical aspect of AKBA’s protective effect lies in its anti-inflammatory action through the inhibition of the HMGB1/TLR4/NF-κB/TNF-α signaling pathway. AKBA also restored cartilage matrix homeostasis by markedly reducing the catabolic enzyme MMP-13 while enhancing the anabolic and protective markers TIMP-1 and SOX9, which was further reflected in the normalization of the cartilage degradation biomarker CTX-II. In addition, the observed inhibition of necroptosis, evidenced by the suppression of the p-RIPK1/p-RIPK3/p-MLKL cascade following AKBA administration, represents another crucial mechanism underlying its protective effect. AKBA modulatory sequel on both miR-34a-5p and miR-146a was also confirmed. Finally, the histopathological results supported the positive biochemical and molecular findings. It is worth mentioning that this research was the first to explore whether AKBA (as a single pure compound at a dose of 250 mg/kg) can provide protection against OA induced by MIA injection. These integrated interactions are summarized in the proposed mechanistic model (Figure 11).

The results revealed that AKBA treatment at a dose of 250 mg/kg attenuated the deleterious morphological changes induced by MIA in cartilage, resulting in a smooth and shiny articular surface resembling that of the sham rats. AKBA prevented the deleterious effects of MIA, preserved the knee joint structure, and maintained a nearly normal histological architecture of the articular surface, as evidenced by the improved modified Mankin scores, characterized by lower surface integrity and cellularity scores comparable to those of the sham-operated group.

MIA administration markedly disrupted cartilage matrix homeostasis, as evidenced by the upregulation of MMP-13, a major collagen-degrading catabolic enzyme (Vincenti and Brinckerhoff, 2002). Conversely, reductions in TIMP-1 and SOX9 expression were also observed. The decline in TIMP-1 and SOX9 reflects the suppression of both protective and anabolic mechanisms essential for cartilage maintenance, whereas the marked increase in MMP-13 indicates enhanced catabolic degradation of type II collagen and proteoglycans (Zhang et al., 2015). Consequently, the degradation of articular cartilage in OA induced by MIA results in the loss of its primary constituents, proteoglycans and type II collagen, rendering them reliable indicators for assessing cartilage metabolism (Duclos et al., 2010). Among these, CTX-II is one of the most widely studied biomarkers representing the degradation products of type II collagen (Duclos et al., 2010).

Interestingly, treatment with AKBA250 reversed these detrimental alterations by significantly reducing MMP-13 and elevating TIMP-1 and SOX9 expression (Zhang et al., 2015). Such modulation indicates that AKBA not only restores the chondrocytic synthetic capacity through SOX9 activation but also restrains matrix degradation by augmenting TIMP-1-mediated inhibition of MMP-13. Supporting our findings, *Boswellia frereana* extract was reported to reduce MMP-13 expression and cartilage matrix breakdown in human articular chondrocytes (Blain et al., 2011). Together, these changes reflect a coordinated protective action of AKBA that re-establishes the balance between anabolic and catabolic cues within the osteoarthritic cartilage. In addition, the beneficial effect of AKBA250 treatment has been demonstrated by normalizing serum CTX-II levels and preventing proteoglycan loss, as confirmed by safranin O stain captured photomicrographs.

The Nrf2/HO-1 pathway is widely recognized as a key protective mechanism against oxidative stress and inflammatory conditions (Ngo and Duennwald, 2022; Saha et al., 2020). Intra-articular injection of MIA resulted in a substantial reduction in Nrf2 and HO-1 protein levels, which is consistent with previous *in vitro* or *in vivo* studies (Cheng et al., 2024; Shi et al., 2022; Busa et al., 2022). Furthermore, in agreement with earlier reports (Jaleel et al., 2020; Lepetsos and Papavassiliou, 2016), the pronounced oxidative state and the decline in the antioxidant defense system led to increased lipid peroxidation and nitrosative stress, as evidenced by elevated MDA and NO levels, respectively, in the untreated MIA group. These elevated oxidative and nitrosative stress markers play a significant role in cartilage degradation and the progression of OA (Watari et al., 2011).

AKBA250 administration demonstrates its antioxidant potential by increasing Nrf2 nuclear translocation, which is reflected in its downstream molecule HO-1 levels, and these results are consistent with previous findings in obsessive-compulsive disorder and experimental autoimmune encephalomyelitis models (Sethi et al., 2023; Nadeem et al., 2022). To the best of our understanding, these findings represent the initial direct evidence of the AKBA’s protective impact in the OA model via the Nrf2/HO-1 antioxidant pathway. AKBA’s antioxidant capability was further confirmed by its ability to counteract the MIA-induced increase in the MDA and NO levels and the decrease in SOD activity, as documented previously (Minj et al., 2021; Chen et al., 2016; Taherzadeh et al., 2022). This could be attributed to the enhancement of glutathione production by Nrf2, which opposes oxidative stress. Additionally, the antioxidant function of HO-1, which prevents lipid peroxidation, arises from its capacity to ultimately generate bilirubin from heme (Gendy et al., 2022).

In addition to oxidative stress, the inflammatory reaction of the chondrocytes also plays a critical role in the development of OA, and it has been linked to the triggering of the body’s innate defense systems through various processes, including TLR activation (Santos-Sierra, 2021). Herein, MIA induced serious inflammatory cascades, which are reflected by the increased contents of knee joint HMGB1, TLR4, NF-κB, and TNF-α. The soluble form of TLR4 has been identified in the synovial fluid of individuals with OA, indicating its potential as a prognostic biomarker (Rabie et al., 2023). Consequently, targeting TLR4 signaling is suggested as a viable therapeutic strategy for treating OA (Bartels et al., 2024). TLR4 has been identified as an NF-κB activator (Gendy et al., 2023). This activation prompts NF-κB to move into the nucleus, as evidenced herein by the increasing nuclear p-NF-κB–p65 expression levels, indicating an enhanced transcriptional activity of inflammatory genes, where it controls the expression of inflammatory cytokines and triggers the deterioration of the articular joint, causing the development and progression of OA (Yao et al., 2023).

In the current study, AKBA250 administration markedly halted the inflammatory response, as revealed by the downregulation of HMGB1 and TLR4 protein expression. This effect may be attributed to the reduction of oxidative stress and enhancement of the Nrf2/HO-1 pathway, as reported herein, where the TLR4 inflammatory response is triggered by different stressors, including oxidative stress (Saha and Rebouh, 2023; Sanada et al., 2021). Another proposed mechanism of TLR4 suppression was suggested to be attributed to Nrf2-mediated anti-inflammatory polarization of macrophages, as these immune cells are the most abundant cells within the synovial joints (Wang and He, 2022). In the same milieu, the elevated levels of the NF-κB protein and the NF-κB nuclear translocation were reduced after AKBA250 administration. As mentioned earlier, this effect could be attributed to the decrease in the upstream modulator TLR4. The results of the previous studies were consistent with our findings (Nadeem et al., 2022; Kundu and Singh, 2023). In addition to the previously mentioned interpretation, the inhibition of NF-κB by AKBA250 can also be attributed to the upregulation of the Nrf2/HO-1 pathway and decrease in MDA and NO levels (Lepetsos and Papavassiliou, 2016; Ahmad et al., 2020; Yin et al., 2009).

This pathway has been previously reported to mitigate the inflammatory role of NF-κB and shift the cellular conditions to a more reducing state, ultimately terminating NF-κB activation. In addition to suppressing this transcription factor, our research has confirmed that AKBA250 efficiently lowers the levels of pro-inflammatory cytokines, including TNF-α. The observed effects strongly indicate the anti-inflammatory properties of AKBA250 in combating MIA-induced OA.

This anti-inflammatory action may also underlie AKBA’s ability to restore cartilage matrix homeostasis since NF-κB is a known transcriptional activator of MMP-13 and a suppressor of SOX9 expression. Consequently, the inhibition of NF-κB signaling by AKBA could simultaneously attenuate inflammation and promote chondrocyte anabolic activity, thereby linking its anti-inflammatory and chondroprotective effects. In agreement, *Boswellia frereana* extract and BA have also been reported to attenuate cartilage destruction primarily through their anti-inflammatory mechanisms (Blain et al., 2011).

The involvement of necroptosis in OA has drawn considerable attention, prompting its investigation in the present study. The necroptotic pathway is characterized by its strong pro-inflammatory nature and is generally regarded as detrimental to tissue integrity. Consequently, inhibition of this pathway holds significant promise as a therapeutic strategy for inflammatory conditions, including inflammation-associated OA (Ye et al., 2023; Liu et al., 2023). It is of particular interest that the present study unveiled a novel finding: AKBA, for the first time, demonstrated the ability to counteract necroptosis as a form of cell death. This was supported by the observed reduction in the expression of p-RIPK1, p-RIPK3, and p-MLKL in the knee joint tissues of rats administered with MIA, along with the alleviation of knee degeneration (Piao et al., 2023). The reversal of necroptosis by AKBA250 may be attributed to its classical antioxidant properties, as observed in this study. Additionally, the Nrf2/HO-1 axis appears to play a role in justifying the observed improvement. This assumption is based on previous research that reported the activation/phosphorylation of RIPK1, RIPK3, and MLKL in the hepatocytes and cardiac tissues of Nrf2 knockdown mice (Lu et al., 2016; Ge et al., 2020). The potential of AKBA250 to inhibit the TLR4/NF-κB pathway and its subsequent target TNF-α should not be neglected. The interaction between TNF-α and TNF receptor-1 triggers the activation/phosphorylation of RIPK1 and RIPK3, facilitating the translocation of p-MLKL to the cell membrane (Jayaraman et al., 2021). The phosphorylated and functional MLKL facilitated the recruitment of ion channels, leading to a substantial influx of Na^+^ and Ca^2+^ ions, which caused cell membrane rupture and necroptosis (Zhou et al., 2022).

Beyond inflammation, NF-κB signaling is closely intertwined with programmed necrosis (necroptosis) as it upregulates several upstream mediators of the RIPK1/RIPK3/MLKL cascade. Meanwhile, NF-κB was documented to transcriptionally regulate components in the RIPK1 pathway. Thus, the observed inhibition of necroptosis following AKBA treatment may partially stem from its ability to suppress NF-κB activation and its associated cytokine release (Du and Wang, 2024).

Nevertheless, it remains uncertain whether the observed modulation of necroptosis results from a direct molecular interaction of AKBA with the necroptosis machinery (RIPK1/RIPK3/MLKL) or reflects an indirect effect secondary to its well-characterized antioxidant and anti-inflammatory actions. This distinction requires further mechanistic investigation in future studies.

In addition to modulating oxidative stress, inflammation, and necroptosis, AKBA appears to exert post-transcriptional regulation through microRNAs that act as fine-tuning regulators of the same signaling networks.

Several miRNAs have been proposed as potential biomarkers for OA as they are involved in chondrocyte proliferation, apoptosis, extracellular matrix metabolism, and inflammation (Veronesi et al., 2023). The selection of miR-34a-5p and miR-146a in this study was based on their well-established mechanistic roles in OA pathogenesis as both regulate chondrocyte apoptosis, inflammatory signaling, and extracellular matrix turnover. Specifically, miR-34a-5p has been reported to be upregulated in osteoarthritic cartilage, where it promotes chondrocyte apoptosis through the phosphatidylinositol-3-kinase/protein kinase B and delta-like ligand 1 pathways. In contrast, miR-146a modulates the TLR4/IL-1 receptor-associated kinase 1/TNF receptor-associated factor 6 signaling axis and nuclear NF-κB-mediated inflammation, thereby linking it directly to cartilage degradation and osteoarthritis progression (Yamasaki et al., 2009).

The functional role of miR-34a-5p in knee OA is very interesting. Endisha et al. (2021) reported that miR-34a-5p expression was increased in the cartilage and synovium of patients with OA. Moreover, miR-34a-5p expression was elevated in the mice’s knees that were fed a high-fat diet. Additionally, intra-articular administration of miR-34a-5p resulted in a pro-inflammatory response, leading to increased expression of key markers associated with knee OA (Caldo et al., 2024). These outcomes are consistent with the miR-34a-5p results in the current study. On the other hand, administration of AKBA250 markedly downregulated miR-34a-5p expression. To the best of our knowledge, this effect on miR-34a-5p was documented for the first time in the current investigation. Furthermore, miR‐34a‐5p antisense oligonucleotide injection was reported to provide joint protection against degeneration effects (Endisha et al., 2021). Abouheif et al. (2010) also reported that silencing miR-34a-5p inhibits chondrocyte apoptosis in both *in vitro* and clinical models (Zhang et al., 2020).

Similarly, previous studies have identified miR-146a as an inflammation-related miRNA that becomes upregulated following cartilage injury and contributes to OA progression through NF-κB activation and cytokine signaling. Suppression of miR-146a expression was shown to protect mice from injury-induced OA and reduce inflammatory damage (Qin et al., 2023). In addition, miR-146a was reported to be overexpressed after injury in human chondrocytes, where silencing miR-146a protects mice against injury-induced OA (Qin et al., 2023). In the current work, AKBA250 administration hindered the elevation in the miR-146a expression. This can be explained partially by inhibiting the NF-κB pathway, as documented in this study and by Shao et al. (2020) and Cheleschi et al. (2019). Moreover, the expression of miR-146a is elevated noticeably by TNF-α stimulation, and the inhibition of this inflammatory cytokine by the current drug will oppose its effect (Li et al., 2012; Yu et al., 2015). It is essential to mention that reducing miR-146a expression may enhance Nrf2 levels, as documented in the current work (Hou et al., 2021). In the current study, the MIA-treated group showed a significant increase in the apoptotic marker caspase-3, which is consistent with the well-established role of apoptosis in MIA-induced OA (Chiu et al., 2016; Jiang et al., 2013). Previous studies have suggested that a high oxidative state, increased reactive oxygen species production, and caspase-3 activation contribute to chondrocyte apoptosis, supporting our present findings.

## 5 Conclusion

The current study reports, for the first time, the potential of oral AKBA (250 mg/kg) to protect against MIA-induced OA, as shown by alleviating edema, normal right-knee diameter measurements, normalized modified Mankin scores, and restored cartilage matrix balance by reducing MMP-13, upregulating TIMP-1 and SOX9, and lowering serum CTX-II levels. Similarly, AKBA orchestrated the HMGB1/TLR4/NF-κB signaling pathway through its anti-inflammatory characteristics. Meanwhile, AKBA’s antioxidant potential was confirmed by increasing Nrf2 and HO-1 contents and reducing MDA levels. In addition, AKBA prominently reduced the protein expression of p-RIPK1, p-RIPK3, and p-MLKL. Furthermore, AKBA exhibited halted miR-34a-5p and miR-146a expression. Together, AKBA demonstrated a protective function in OA by inhibiting inflammatory signals through the TLR4/NF-κB pathway, augmenting the cytoprotective Nrf2/HO-1 pathway, and modulating the necroptosis signaling cascades. Collectively, these actions highlight AKBA’s multifaceted chondroprotective potential and support its development as a candidate therapy to delay the onset and the progression of OA.

## 6 Limitations and future directions

Despite these comprehensive findings, the study lacks direct mechanistic interventions to confirm the causal roles of the examined pathways. The observed modulation of NF- κB, Nrf2, necroptosis-related proteins, and miRNAs by AKBA provides strong associative evidence but no definitive cause–effect link. Future studies using targeted inhibition, gene silencing, or overexpression are necessary to substantiate AKBA’s mechanistic role. Similarly, miRNA overexpression or inhibition approaches are needed to verify the direct involvement of miR-34a-5p and miR-146a. Moreover, *in vitro* cellular studies should be conducted to confirm the proposed signaling interactions underlying AKBA’s chondroprotective and anti-inflammatory actions.

The MIA-induced OA model, characterized by rapid chondrocyte death, may not fully replicate the gradual, mechanically driven pathology of post-traumatic OA. Nonetheless, these findings provide valuable preclinical evidence with potential translational relevance. Only male rats were used to minimize hormonal variability; future studies should include both sexes to clarify possible gender-related differences. The AKBA dose of 250 mg/kg/day used herein corresponds to a human equivalent dose of ∼40 mg/kg (approximately 2.4 g/day for a 60-kg adult), which lies within the reported safety margins (Du et al., 2015). Although such levels are unattainable through raw Boswellia extracts (∼2%) (Saadh et al., 2020), they may be achievable using standardized or bio-enhanced formulations (10%–30% AKBA) (Mamatha et al., 2025).

## Data Availability

The original contributions presented in the study are included in the article/Supplementary Material, further inquiries can be directed to the corresponding author.

## Abbreviations

AKBA, 3-O-Acetyl-11-keto-β-boswellic acid; ANOVA, one-way analysis of variance; BAs, boswellic acids; CTX-II, crosslinked C-telopeptide of type II collagen; DAMPs, damage-associated molecular patterns; ELISA, enzyme-linked immunosorbent assay; H&E, hematoxylin and eosin; HMGB1, high mobility group box 1; HO-1, heme oxygenase-1; i.a, intra-articular; MDA, malondialdehyde; MIA, monosodium iodoacetate; miRs, microRNAs; MLKL, mixed lineage kinase domain-like protein; NF-κB, nuclear factor kappa B; Nrf2, nuclear factor erythroid 2-related factor-2; OA, osteoarthritis; PBS, phosphate buffered saline; qRT-PCR, quantitative real-time polymerase chain reaction; RIPK1, receptor-interacting protein kinase 1; RIPK3, receptor-interacting protein kinase-3; SD, standard deviation; SOD, superoxide dismutase; MMP-13, matrix metalloproteinase-13; SD, standard deviation; SOX-9, SRY-box transcription factor 9; TIMP-1, tissue inhibitor of metalloproteinase; TLR4, toll-like receptor 4; TNF-α, tumor necrosis factor-α.
