# Supplementary material for: Acetyl-11-keto-beta-boswellic acid ameliorates monosodium iodoacetate-induced osteoarthritis in rats: implications of HMGB1/TLR4/NF-κB and Nrf2/HO-1
Source: Front Pharmacol. 2025 Dec 1;16:1694803. doi: 10.3389/fphar.2025.1694803 (PMC12702862; doi:10.3389/fphar.2025.1694803)
Supplement: Supplementary file 1 [file DataSheet1.docx]

**Supplementary data:**A preliminary dose-probing investigation was performed to compare the efficacy of AKBA 125 mg/kg and AKBA 250 mg/kg in MIA-induced osteoarthritis (OA) rats. The AKBA 250 mg/kg dose was subsequently selected for the main study due to its superior biochemical and histological outcomes. The two doses were chosen based on previous reports (Barakat et al., 2018; Singh et al., 2007; Tawfik, 2016) and guided by our recent findings on AKBA in repetitive traumatic brain injury (El-Gazar et al., 2024). Both AKBA doses markedly reduced joint swelling, restoring knee diameter values to near-normal levels (**S.Fig.1-I**). Macroscopic examination confirmed that AKBA treatment ameliorated the morphological damage induced by MIA, with the 250 mg/kg dose showing greater improvement, yielding a smooth, shiny articular surface comparable to sham controls (**S.Fig.1-II**).Histologically, both doses preserved cartilage structure, while AKBA 250 mg/kg demonstrated superior protection against cartilage degeneration, (**S.Fig.1-IV**). Safranin O staining revealed that AKBA125 partially reduced proteoglycan loss, whereas AKBA250 restored normal matrix staining and prevented cartilage depletion.Biochemically, AKBA 250 mg/kg exerted a more pronounced normalization of serum CTX-II and joint TIMP-1 expression (**S.Fig.1-III**), supporting its stronger chondroprotective and anti-inflammatory effects.Based on these findings, AKBA 250 mg/kg was selected as the effective therapeutic dose for subsequent mechanistic investigations.


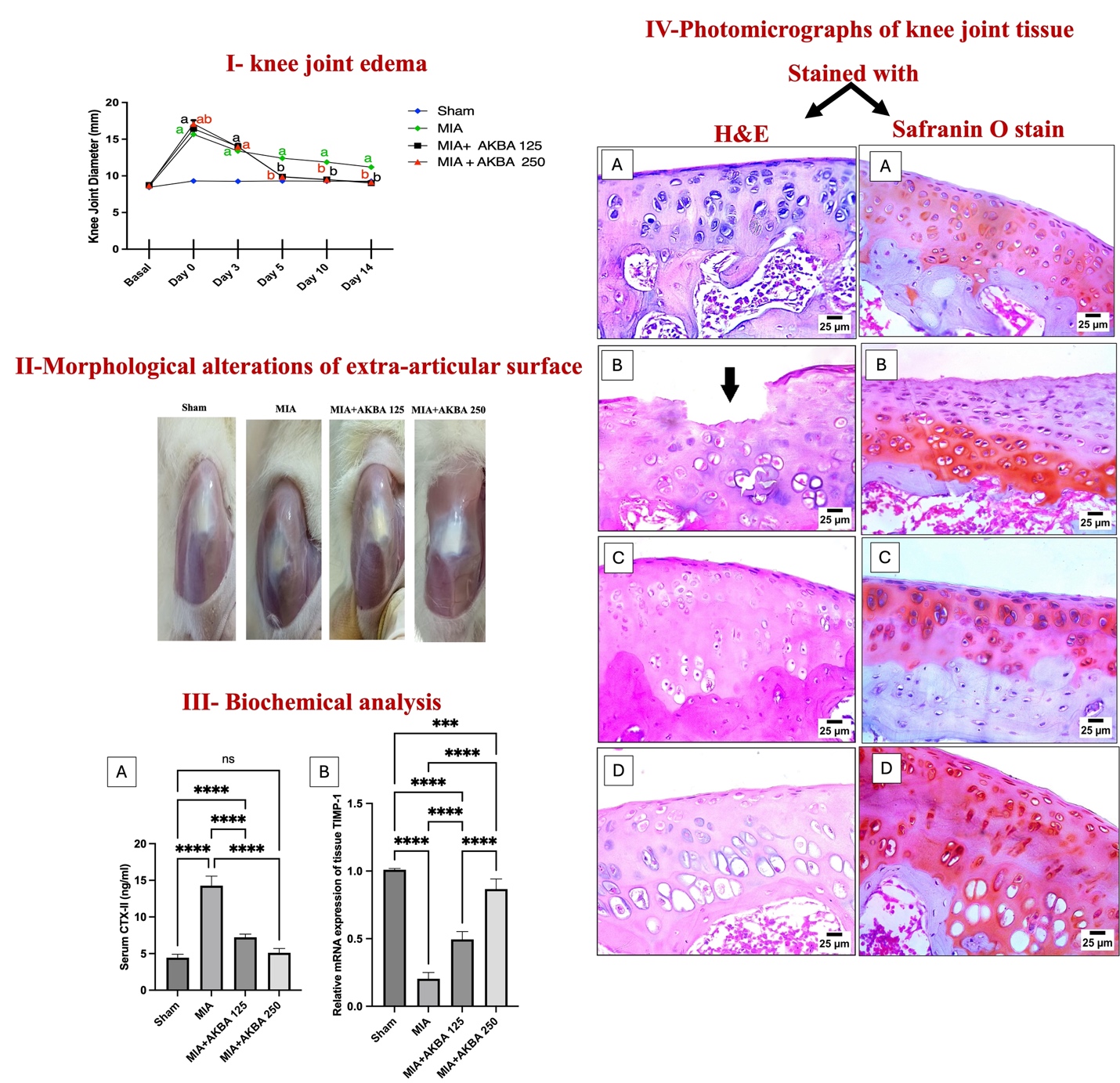


**S.Fig.1. Findings of the dose-probing investigation of the effective AKBA dose against MIA-induced osteoarthritis in rats.(I) Knee joint edema: Digital caliper assessment of joint diameter (mm) across experimental groups. The symbol (a) indicates a significant difference from the sham group, while (b) indicates a significant difference from the MIA-only treated group. Data are presented as mean ± SD (n = 9) and analyzed using two-way repeated measures ANOVA with one factor repetition (time: day); P < 0.05 was considered significant.(II) Morphological alterations of the extra-articular surface: The sham group displayed a normal, smooth, and shiny surface; the MIA group exhibited dull yellowish discoloration, whereas AKBA125 and AKBA250 treatments restored a smoother, glossier surface.(III) Biochemical analysis: (A) Serum CTX-II levels and (B) knee joint TIMP-1 expression. Data are expressed as mean ± SD (n = 6), analyzed using one-way ANOVA followed by Tukey’s post hoc test. The number of asterisks above the columns indicates the degree of significance (P < 0.05, P < 0.01, P < 0.001, P < 0.0001). (IV) Histological evaluation: Photomicrographs of knee joint tissues stained with Hematoxylin & Eosin (H&E, left panels) and Safranin O (right panels). Panels A–D and A–D** represent sham, MIA, MIA + AKBA125, and MIA + AKBA250 groups, respectively. Abbreviations: AKBA, 3-O-Acetyl-11-keto-β-boswellic acid; MIA, monosodium iodoacetate; CTX-II, crosslinked C-telopeptide of type II collagen; TIMP-1, tissue inhibitor of metalloproteinase-1; SD, standard deviation.**

Barakat, B. M., Ahmed, H. I., Bahr, H. I., & Elbahaie, A. M. (2018). Protective Effect of Boswellic Acids against Doxorubicin-Induced Hepatotoxicity: Impact on Nrf2/HO-1 Defense Pathway. *Oxidative Medicine and Cellular Longevity*, *2018*. https://doi.org/10.1155/2018/8296451

El-Gazar, A. A., El-Emam, S. Z., M. El-Sayyad, S., El-Mancy, S. S., Fayez, S. M., Sheta, N. M., Al-Mokaddem, A. K., & Ragab, G. M. (2024). Pegylated polymeric micelles of boswellic acid-selenium mitigates repetitive mild traumatic brain injury: Regulation of miR-155 and miR-146a/BDNF/ Klotho/Foxo3a cue. *International Immunopharmacology*, *134*. https://doi.org/10.1016/J.INTIMP.2024.112118

Singh, S., Khajuria, A., Taneja, S. C., Khajuria, R. K., Singh, J., & Qazi, G. N. (2007). Boswellic acids and glucosamine show synergistic effect in preclinical anti-inflammatory study in rats. *Bioorganic & Medicinal Chemistry Letters*, *17*(13), 3706–3711. https://doi.org/10.1016/J.BMCL.2007.04.034

Tawfik, M. K. (2016). Anti-aggregatory effect of boswellic acid in high-fat fed rats: involvement of redox and inflammatory cascades. *Archives of Medical Science : AMS*, *12*(6), 1354–1361. https://doi.org/10.5114/AOMS.2016.60675
